# Digital child protection in social networks: age verification and age-tiered regulation in Europe

**DOI:** 10.1186/s13034-025-01016-x

**Published:** 2025-12-23

**Authors:** Franziska Köhler-Dauner, Lena Peter, Emily Sitarski, Katrin Chauviré-Geib, Ann-Christin Haag, Jörg M. Fegert

**Affiliations:** 1 Department of Child and Adolescent Psychiatry, Psychosomatics and Psychotherapy, University Hospital of Ulm, Steinhövelstraße 5, 89075 Ulm, Germany; 2 German Center for Mental Health (DZPG), Partner Site Mannheim-Heidelberg-Ulm, Mannheim, Germany; 3 Competence Area Mental Health Prevention in the Competence Network Preventive Medicine Baden-Württemberg, Ulm, Germany; 4 Competence Center Public, Child Mental Health, Partner Site Ulm, Steinhövelstraße 5, 89075 Ulm, Germany

## Introduction

The digital lifeworld of children and adolescents has become a central component of socialisation, education, and identity formation. Digital spaces are no longer an add-on, but a structural element of contemporary childhood. Social media enables communication, agency, and a sense of belonging, yet at the same time confront children with substantial risks: sexualised approaches, cyberbullying, algorithmic manipulation, commercialisation of personal data, and disinformation have become defining dimensions of endangerment [1–3].

Governments around the world are responding with differing strategies. In November 2025, the European Parliament added further momentum to this global trend by proposing to raise the minimum age for accessing social media to 16, combined with mandatory, privacy-preserving age-verification obligations for platforms [4]. In 2024, Australia introduced the widely noted Social Media Minimum Age Act, which prohibits children under 16 from accessing social networks and obliges platforms to implement mandatory age-verification systems [5]. France, Norway and several US states are pursuing similar, restrictive approaches. The United Kingdom, by contrast, has adopted a child-rights-based regulatory framework and technical duties of care with its Age-Appropriate Design Code, while Canada combines preventive education and reporting schemes with data protection [6, 7].

This heterogeneity points to a shared dilemma: pure bans or access restrictions fall short. They impede the development of digital competences, create social inequalities, and encourage circumvention strategies. Empirical studies show that children can easily bypass age limits and thereby unintentionally migrate into less regulated, higher-risk digital environments [2, 8–10]. This position is echoed in the recent statement by the Swiss EKKJ, which emphasises that general social-media bans are ineffective and hinder essential developmental and participatory processes [11]. Exclusion from social media also entails exclusion from central spaces of social participation, particularly for children in vulnerable life circumstances. A policy that relies solely on avoidance thus reinforces inequalities and shifts risks rather than mitigating them [2, 6, 12].

This policy paper therefore argues for regulated, age-tiered participation as a realistic and socially balanced alternative to blanket prohibitions. The policy paper outlines the current challenges in digital child protection, compares international regulatory approaches, and critically evaluates age-verification and age-tiered participation models. It concludes with policy recommendations that balance child protection with participation rights and digital freedoms. Age verification and differentiated access levels are intended to ensure that children are protected in line with their developmental stage, without being excluded. A tiered model envisages that children under the age of ten have no access to social media, children between ten and thirteen interact in moderated, pedagogically supported environments and adolescents from the age of thirteen participate in regular platforms under verified conditions [6, 7]. The aim is a balanced model of child protection that minimises risks without undermining the opportunities offered by digital participation.

## Initial situation and problem definition

Over the past two decades, digitalisation has evolved from a technological innovation into a defining lifeworld of childhood and adolescence. Children and young people now grow up in digital environments that are not only spaces of communication, but also contexts of learning and socialisation. Empirical studies demonstrate that digital media are no longer experienced as a mere complement to the analogue world but as an integral part of it [2, 7]. Within this context, social networks function as platforms for identity formation, relationship maintenance and participation in public discourse [3]. They enable children to express emotions, experiment with self-images and actively participate in public communication processes. Digital spaces have thus become a constitutive element of modern childhood.

This development is accompanied by a profound restructuring of traditional developmental contexts. Digital platforms are increasingly assuming functions that were previously fulfilled by family, school or local communities. They offer recognition, social resonance and orientation, while simultaneously acting as mediators of values and norms [6, 7]. The EKKJ similarly stresses that children require opportunities to practise digital skills within supported environments rather than being excluded from them [11]. Excluding children and adolescents from social media would therefore, for many of them, mean exclusion from central forms of social interaction, cultural affiliation and informal learning. This aligns with recent statements by the EKKJ, which underline that children’s rights also apply in digital environments, including their rights to participation, access to information and involvement in decisions affecting them [11]. In this sense, social media are not only a source of risk, but also a precondition for social participation [2, 12].

At the same time, the digital lifeworld of children and adolescents is characterised by a significant concentration of risks. Children are increasingly confronted with sexualised contact, cyberbullying and algorithmically steered attention-capture [6, 13]. The commercialisation of personal data opens up new forms of economic exploitation, while algorithmic recommendation systems prioritise content that intensifies emotions and maximises interaction [14]. As a result, children find themselves in an environment shaped less by chance than by deliberate design: their perceptions, preferences and social relationships are increasingly governed by algorithmic structures whose functioning remains largely opaque to them [7, 15].

The digital lifeworld of children is by no means homogeneous, but varies significantly by age and developmental stage. Children in early primary school (up to around nine years of age) typically have limited ability to assess digital risks and display a higher level of trust in media-mediated content that they obtained by themselves [16]. Studies show that children in this age group tend to perceive virtual communication partners as equivalent to real ones and find it difficult to recognise commercial or manipulative intentions [10]. This group is therefore particularly dependent on structural protection mechanisms, technical access restrictions and pedagogical support [6, 7].

Children aged ten to thirteen are vulnerable as well. From a developmental-psychological perspective, this phase marks a transition between cognitive and emotion regulation immaturity and increasing social orientation. Research has shown that children at this age tend to underestimate risks and react strongly to social feedback, particularly recognition from peers [17]. This combination of exploratory behaviour and limited risk competence makes them particularly susceptible to problematic online experiences, offender contact or manipulative platform mechanisms. At the same time, digital interactions become increasingly important for identity development and group affiliation in this age group, which can intensify emotional dependence on online communication [3].

By contrast, adolescents from around fourteen years of age exhibit more advanced cognitive and meta-reflective capacities. They are increasingly able to recognise risks in abstract terms, to develop their own protective strategies and to critically assess the social context of digital interactions [14] and digital literacy also tends to increase with increasing age [18]. However, adolescents encounter a range of online risks [19] and exposure to forms of endangerment rises in this phase, particularly through algorithmically amplified ideological content, political polarisation and addiction-like patterns of social media use [15]. Accordingly, the focus of protection needs shifts from structural to educational measures targeting media literacy, self-regulation and critical reflection.

Existing protection mechanisms are so far insufficient to meet these challenges. At a European level, the Digital Services Act (DSA) formulates due-diligence obligations for platforms, but does not provide harmonised rules for age verification [5]. The 2025 European Parliament proposal illustrates this tension: while aiming to strengthen protection by raising the minimum age to 16, it risks reinforcing a paternalistic logic if not combined with empowerment-oriented educational measures and child-rights-based design principles [4]. National regulations, such as the UK’s Age-Appropriate Design Code or Australia’s Enhancing Online Safety for Children’s Act 2015, demonstrate that platform responsibility can be legally enforced, yet there remain considerable differences in interpretation, implementation and oversight [13]. In many member states it is unclear which authority is ultimately responsible for monitoring and sanctions. Furthermore, technical standards for secure, data-protection-compliant age verification are lacking.

These regulatory deficits are compounded by social inequalities in digital participation. Studies show that children from socio-economically disadvantaged families, with a migration background, with mental health burdens or histories of childhood adversities are more frequently exposed to digital risks and at the same time benefit less from preventive protection measures [2, 3, 20, 21]. For these children, social media may constitute vital spaces to overcome isolation and access support. Restrictive access bans would exclude precisely these groups from digital communication and expression opportunities from which they particularly benefit.

This intensifies the tension between the state’s legitimate duty to protect and the right to digital participation. While one side emphasises the responsibility to shield children from potential harm, the other highlights the importance of digital integration for education, equality of opportunity and social belonging [14]. Thus, the central dilemma is not whether children should be protected, but how this protection can be designed without undermining participation opportunities.

With this background, this policy paper aims at developing a model of regulated participation that links legal regulation, technical age verification and pedagogical support. Only a multidimensional perspective can produce a sustainable and child-appropriate protection framework that takes children’s lifeworlds seriously and ensures both safety and social participation.

## International perspective and current regulatory approaches

Internationally, the regulation of digital child protection is characterised by a high degree of diversity in national approaches. Despite similar underlying challenges—namely the tension between safety, data protection, and participation—legal and institutional responses vary considerably. This heterogeneity reflects a shared dilemma: to date, no coherent global or European standards exist that integrates age verification, children’s rights, and media-educational approaches [5]. While some countries have adopted restrictive access limitations, others pursue models grounded in awareness-raising and platform accountability. The following section analyses the regulatory logics of Europe, Australia, Canada, and the United States, in order to evaluate their potential and limitations for the development of a European model of regulated participation.

Within Europe, there are significant disparities in the regulation of social media for minors. Most recently, the European Parliament called for a legally binding EU-wide minimum age of 16 for social-media use, accompanied by mandatory, privacy-preserving age-verification requirements and reinforced safety-by-design obligations [4]. Tthe *Digital Services Act* (DSA) constitutes the primary legal framework. It obliges platforms to ensure transparency, conduct risk assessments, and moderate content, yet it contains no binding provisions on age verification. Consequently, member states have developed their own, often divergent, strategies [5].

The United Kingdom is regarded as a pioneer of child-rights-oriented digital policy. With the *Age-Appropriate Design Code* (AADC), enshrined in the *Data Protection Act 2018*, a legally binding framework was established that requires digital service providers to design products that are “child-appropriate.” The AADC is based on the principle of *privacy by design*: platforms must minimise data processing, set default configurations to maximise privacy, and ensure age-appropriate communication formats. This model is internationally recognised as a best-practice example because it combines legal, technical, and ethical dimensions [22]. Evaluation reports from the UK Information Commissioner’s Office (ICO) show that, following the introduction of the Code, the proportion of child-friendly privacy settings on major platforms increased by 45%, while risky contact options declined by 30% [5, 23].

France, by contrast, adopted a law in 2023 that mandates compulsory age verification for social media platforms and requires parental consent for children under the age of 15. The objective is to protect minors from harms such as grooming and cyberbullying. According to a representative survey of French families, 68% of parents support this regulation, while 54% of adolescents report being able to circumvent it through falsified age information [24]. Legal scholars, however, criticise this approach for overemphasising parental control while neglecting children’s rights—particularly their rights to information and participation [5].

Norway has similarly announced plans to raise the minimum age for social media use from 13 to 15 years, allowing parents to authorise exceptions for younger children. Both France and Norway exemplify a broader trend towards more restrictive age limits, which, however, generate normative tensions between protection and autonomy.

In Germany, the *Interstate Treaty on the Protection of Minors in the Media* (*Jugendmedienschutz-Staatsvertrag*, JMStV) provides the legal foundation. It obliges providers to label content that may impair development and to implement access restrictions. In practice, however, implementation remains fragmented due to federal responsibilities, the lack of clear age-verification standards, and an overemphasis on content rather than platform architectures. Evaluation data from the Commission for the Protection of Minors in the Media [25] indicate that fewer than half of platforms apply age classifications correctly, and violations are rarely sanctioned. While the JMStV strongly emphasises the protective dimension, it lacks binding rules on structural safety or algorithmic transparency [26].

In summary, European countries tend to oscillate between two poles: on the one hand, restrictive access models emphasising protection and parental authority; on the other, rights-based approaches focusing on child-appropriate design and digital agency. A unified European standard has yet to be developed.

Australia is widely recognised as a pioneer in institutionalised online child protection. The *Enhancing Online Safety for Children’s Act 2015* established the *Office of the eSafety Commissioner*—an independent authority endowed with broad powers for education, training, and enforcement of content removal orders [22]. Since 2015, more than 15,000 cyberbullying complaints have been processed, and over 80% of reported content has been removed within 48 h [13]. This system is complemented by initiatives such as *Stay Smart Online* and school-based education programmes promoting safe internet use. With the *Social Media Minimum Age Act 2024*, Australia significantly tightened its regulation: children under 16 are prohibited from accessing social networks, and platforms must verify user identities or face substantial fines. The stated aim is to protect children from online harms and to strengthen parental oversight. Critics such as Stalford and Lundy [5] view this as a paternalistic strategy that restricts children’s rights to participation and expression. Concerns have also been raised regarding the practicality and data protection compliance of age-verification systems. Initial evaluations by the eSafety Office [27] show that 41% of affected adolescents attempt to circumvent the ban through alternative platforms or VPNs, thereby increasing their exposure to unregulated online spaces. The Australian approach thus exemplifies the ambivalence of restrictive policy: while it enhances platform accountability, it simultaneously risks excluding children who rely on social media for education and social connection. Nevertheless, Australia remains a key example of the institutional effectiveness of state-led online safety structures.

Canada follows a preventive-technological approach. The *Canadian Centre for Child Protection* (C3P), a national non-profit organisation, coordinates protective measures and operates *Cybertip.ca*, a central reporting hub for online exploitation. In addition to this reactive component, a wide range of educational initiatives exists: *ProtectKidsOnline.ca* provides parents with age- and risk-specific information, while *Zoe and Molly Online* and *NeedHelpNow.ca* target children and adolescents [22]. According to C3P’s 2024 Annual Report [28], the use of these programmes increased by 35%, and 92% of participating schools reported improved student awareness of online risks. Technologically, the project *Project Arachnid* is particularly innovative: using algorithmic web crawlers, it identifies and blocks the dissemination of child sexual abuse material worldwide. By 2023, over 10 million URLs had been analysed and more than 7 million images removed [29]. Canada thus combines technological intervention with education and legal oversight. However, it has not introduced age limits or mandatory verification systems. Instead, it relies on self-regulation and voluntary platform cooperation. This model reflects a preventive and educational approach that views learning as a form of protection, though it is less structurally binding than the European or Australian systems. Empirical long-term studies show that Canadian adolescents score above the international average in media literacy [6].

The United States follows a primarily consumer-protection-oriented digital policy. The *Children’s Online Privacy Protection Act* (COPPA) of 1998 requires service providers to obtain parental consent before collecting personal data from children under 13. The focus of this framework is therefore on data protection rather than algorithmic safety or media education [15].

In recent years, several US states have adopted more restrictive legislation. The *Utah Minor Protection in Social Media Act* (2023) required parental consent for users under 18 but was struck down as unconstitutional for violating freedom of expression. According to the *Center for Democracy & Technology* [30], over 60% of affected adolescents continue to maintain social media accounts despite legal restrictions. In Florida, where a general ban for under-14 s was introduced in 2025, no significant decline in problematic usage patterns has been observed [15]. Scholars argue that this focus on parental control and consumer protection is structurally flawed: children are treated as passive objects of protection, and their rights to privacy, expression, and participation remain largely disregarded [2]. This tendency illustrates that, unlike Europe, the United States places emphasis on individual responsibility and parental authority rather than on state-coordinated protection systems—marking a fundamental divergence from European children’s rights frameworks.

A comparative international perspective reveals three key trends. First, there is a pronounced tension between restrictive prohibitionist policies and participatory protection models. Countries such as Australia, France, and parts of the United States rely primarily on age-based access restrictions, whereas the United Kingdom and Canada emphasise design obligations, educational strategies, and institutionalised platform accountability. Cross-national studies demonstrate that the latter approaches achieve more sustainable outcomes: programmes promoting digital competence and safe online behaviour have led to significant reductions in cyberbullying and grooming incidents in both Canada and the UK [2, 15].

Second, international evaluations indicate that restrictive age limits are largely ineffective in practice. Children and adolescents often circumvent them through technical means or alternative accounts, resulting in little or no actual reduction of risk exposure [2, 22]. In fact, problematic use tends to shift toward less regulated environments, thereby generating new forms of risk [13].

Third, empirical findings suggest that child-rights-based and education-oriented models yield the most effective and sustainable outcomes. These systems combine legal responsibility with pedagogical empowerment, promoting digital self-efficacy rather than control. Protection is thus conceptualised as enabling rather than restricting—a paradigmatic shift supported both empirically and normatively in line with the UN Convention on the Rights of the Child [5].

For Europe, these findings imply a clear mandate: instead of continuing along paths of national fragmentation, a comprehensive, integrated model should be developed—one that combines binding age verification, safety-by-design obligations, and educational integration. Only such an evidence-based governance approach can sustainably balance safety, data protection, and social participation.

## Regulatory options: age verification and age-tiered participation

The analysis of international experience shows that effective digital child protection must aim at regulated participation rather than general prohibitions. Empirical evidence demonstrates that access restrictions alone neither solve problems nor significantly reduce them; instead, they frequently lead to rule-evasion and thus generate new risks [22, 27]. Furthermore, parental monitoring seems to be more protective against negative effects of internet usage such as online harassment than parental internet restriction [31]. At the same time, regulated participation does not preclude clear age-based prohibitions where developmental considerations demand it. In particular, a categorical exclusion of younger children from social media must be understood as a central pillar of any coherent protection regime, which becomes feasible only if it is tied to robust and enforceable age-verification systems. In consequence, the debate on technically robust age-verification systems and age-tiered concepts of use has gained importance.

Age verification constitutes the technical core instrument of a differentiated protection system. The European Parliament’s 2025 proposal aligns with this need for robust verification mechanisms: the draft regulation explicitly requires platforms to implement mandatory, privacy-preserving age-verification technologies as a prerequisite for enforcing the proposed minimum age of 16. This underscores the wider recognition in European policymaking that age-tiered access models depend on technically reliable identification systems [4]. Its aim is to hold platforms accountable for reliably verifying users’ actual ages without infringing data protection or informational self-determination. Various methods are discussed in the literature, including digital identity credentials, biometric procedures and analogue models linked to existing structures such as mobile phone contracts or school IDs [5, 26]. What matters is not only technical feasibility but also normative orientation: from a child-rights perspective, age verification should be designed as a prerequisite for both the strict prohibition of access for younger children and the regulated inclusion of older age groups. It should not become a tool of generalized surveillance, but rather a means of enabling safe and age-appropriate participation along clearly defined age thresholds. An effective protection system must embed this technical component in an age-tiered regulatory model guided by developmental-psychological and social parameters. Empirical findings from child and adolescent psychology confirm that digital competence and risk processing change with age and therefore require a graduated approach [14, 26]. This yields the following framework:

1. **Early Childhood (0–9 years):** In this phase, protection needs are particularly high. Children do not yet possess the cognitive, emotional and social competences required to critically evaluate digital interactions. Against this background, a legal and practical prohibition of social media accounts for this age group is indicated. Accordingly, access to social media should remain forbidden. Instead, protected, pedagogically curated digital learning and communication environments should be provided, offering child-appropriate content and safe interaction [10]. Such a prohibition for under-10 s can only be effective if platforms are obliged to implement stringent age-verification procedures that reliably prevent the creation of accounts for this group.
2. **Preadolescence (10–13 years):** This age group is particularly vulnerable, as exploratory behaviour coincides with limited risk competence. Research shows that children in this phase benefit most from guided digital use [26]. The EKKJ also highlights the importance of guided learning processes, emphasising that media competence emerges through active engagement, reflection and supportive adult involvement [11]. As a result, access to social media should not be banned but structurally limited—for example through “school versions” of social networks that rely on contact restrictions, algorithmic safeguards and pedagogical moderation [15]. Within such environments, children can develop digital competences without being fully exposed to the risks of commercial platforms. In regulatory terms, this implies that standard commercial versions of social media remain inaccessible to this age group, while access to specially designed, education-embedded environments is explicitly permitted and regulated.
3. **Adolescence (13–16/17 years):** With increasing cognitive maturity and growing social responsibility, the use of social media may be expanded, provided it takes place under conditions of mandatory age verification and youth-appropriate algorithms. Introducing compulsory age checks—analogous to SIM-card registrations—would ensure that platforms correctly identify minors and afford them enhanced data protection [5]. In addition, safety-by-design mechanisms are needed that prevent manipulative algorithms and provide user-friendly control options [22]. For this age group, regulation therefore shifts from categorical exclusion to conditional access, with age verification functioning as the gatekeeper between prohibited and permitted forms of participation.
4. **Late Adolescence (from 16/17 years):** From this age onwards, a gradual transition to full use can take place. A prerequisite, however, is the integration of digital education into formal and non-formal learning contexts, in order to foster self-regulation, data-protection skills and critical thinking [2]. The transition to unrestricted use should thus be based not only on age but also on competence.

The proposed model explicitly takes into account the social inequalities shaping access to safe digital participation. Children from socio-economically disadvantaged families or vulnerable life situations often use social media as their primary spaces for communication and self-expression [3]. A blanket ban would disproportionately affect these children and deprive them of arenas of self-efficacy and social support. By combining a strict prohibition of social media for younger children with regulated and safeguarded access for older age groups, the model seeks to balance developmental protection with the imperative of social inclusion. Regulated participation—accompanied by pedagogical and technical protection systems—ensures instead that protection does not lead to exclusion, but functions as an instrument of equal opportunity.

International empirical evidence supports this perspective: children who engage in age-tiered digital environments with clear safety and feedback mechanisms are demonstrably less exposed to risks and simultaneously develop higher levels of digital self-efficacy [15]. This shows that child protection and participation are not opposites, but mutually reinforcing within an appropriate regulatory framework.

From a European perspective, this model offers the opportunity to harmonise the fragmented protection architecture of the member states. A unified regulation on age verification, combined with binding requirements for platform design and educational integration, would clearly allocate responsibilities for protection while upholding children’s rights to information and participation. Central to this framework is the explicit legal prohibition of social media use for younger children, implemented through effective age verification, and the subsequent transition to regulated forms of participation for older minors. In doing so, it would facilitate a shift from prohibition-driven policymaking to an evidence-based governance model that links protection, education and digital justice.

## Effectiveness of restrictive regulatory approaches in digital child protection: a critical evaluation

In numerous countries, the introduction of restrictive age limits and access restrictions is regarded as the primary instrument for protecting minors in the digital sphere. The political appeal of such measures lies in their apparent clarity and symbolic impact: they project governmental capacity to act in response to the risks of social media. From a scholarly perspective, however, prohibition strategies prove to be empirically inadequate and normatively ambivalent.

First, several international studies demonstrate that statutory age restrictions are routinely circumvented in practice. In Norway, more than 80 percent of under-15-year-olds maintain a social-media account despite being formally excluded [22]. Similar findings exist for Australia, where approximately 40 percent of adolescents continue to actively use social networks after the Social Media Minimum Age Act 2024 entered into force, often via VPN services or alternative platforms [13]. In France, more than half of respondents reported evading age-verification systems by providing false information [24]. Comparable patterns are observed in the United States: evaluations of the Utah Minor Protection Act and similar state laws show that more than 60 percent of adolescents remain active despite formal bans [2].

This evidence points to a structural enforcement deficit inherent in restrictive policies. The European Parliament’s recent call for a minimum age of 16 demonstrates the political appeal of prohibitionist approaches. Yet, the evidence reviewed in this paper suggests that such measures risk replicating the same enforcement deficits observed in other jurisdictions, unless accompanied by educational, structural and design-based interventions. Age limits that are technically easy to evade do not lead to a significant reduction in digital risks; instead, they shift problematic use into unregulated, opaque online spaces [5, 13]. Prohibitions thus transform from protection mechanisms into risk multipliers: by restricting participation without enhancing safety, they displace endangerment into areas beyond institutional oversight.

Second, developmental-psychological and media-educational research indicates counterproductive psychological effects. Restrictive measures trigger reactance processes among children and adolescents – defensive responses to perceived restrictions of freedom [1]. Empirical studies show that exclusion and paternalism correlate with increased covert use, a greater propensity for risky behaviour and the selection of less secure communication channels [2, 26]. In this way, prohibitions reinforce precisely those practices they aim to prevent. The symbolic and social allure of the “forbidden” heightens its attractiveness, potentially exacerbating risk-taking behaviour.

Third, blanket bans are socially selective and exclusionary. Empirical analyses reveal that children from socio-economically disadvantaged or psychosocially strained backgrounds use social media particularly frequently as primary spaces of communication and self-representation [3]. When these spaces are closed off through restrictive measures, secondary dynamics of exclusion arise: those who already face limited educational and participation opportunities are additionally marginalized in digital terms. Such measures thus conflict with the objectives of digital inclusion and educational equity.

Fourth, a normative inconsistency in restrictive policy can be identified. By conceptualising children primarily as objects of protection, their status as active subjects and rights-holders is overlooked. The UN Convention on the Rights of the Child guarantees rights to freedom of expression, information and participation (Arts. 12 and 13 UNCRC). Prohibition strategies that rely on control instead of empowerment therefore sit uneasily with a child-rights-based understanding of digital policy [5].

Overall, restrictive age limits emerge as neither empirically effective nor normatively coherent. Their political symbolism masks both their factual ineffectiveness and their socio-pedagogical side effects. In contrast, comparative evaluations from the UK and Canada show that combined systems of age verification, design obligations and media-educational programming achieve significantly higher protective impact. Programmes for digital competence development have demonstrably reduced incidents of cyberbullying and grooming while simultaneously enhancing young people’s perceptions of their own agency [2, 28].

This evidence underlines the need for a paradigm shift in digital child protection: away from reactive prohibitions toward a preventive-empowering approach integrating legal, technological and pedagogical components. Protection should not be construed primarily as exclusion, but as the enabling of safe and equitable participation in the digital realm.

## Policy recommendations

The preceding analysis demonstrates that effective child protection in the digital sphere can only be achieved by combining legal, technical and educational approaches. Pure prohibition strategies fall short because they ignore the structural causes of digital risks and reinforce social exclusion. Instead, an evidence-based protection model is required that integrates the principles of age verification, safety-by-design and media education into a coherent governance framework.

At the regulatory level, the first step is to introduce a harmonised European framework for age verification. The European Union’s Digital Services Act (DSA) provides an important foundation, but lacks precise provisions on age control and child-appropriate platform design. The European Parliament’s proposal to set a 16-year minimum age for social-media use highlights the urgency of establishing harmonised European standards. However, its effectiveness will depend on embedding such age thresholds within a broader governance framework of privacy-preserving age verification, safety-by-design obligations and comprehensive media-education strategies. Divergent national standards result in legal fragmentation and hamper the enforcement of effective protection measures [5]. A unified system should define technical, data-protection and ethical requirements. Potential models include interoperable digital identity credentials, school-based authentication procedures or verified mobile-phone solutions, which—in line with the privacy-by-design principle—reliably verify age without retaining disproportionate amounts of personal data. Responsibility for proof of users’ ages would thus lie squarely with platforms, while children’s informational self-determination would be preserved.

This must be supplemented by an obligation to age-adaptive platform design. The UK’s Age-Appropriate Design Code has empirically demonstrated the effectiveness of statutory design obligations: following its introduction, the proportion of safe privacy settings and contact-limiting mechanisms increased significantly [23]. A comparable EU-wide approach could compel platforms to avoid manipulative recommendation algorithms, display safety warnings prominently and configure interaction options in an age-appropriate manner. This would shift the focus from individual misconduct to the structural responsibility of providers.

To ensure and monitor such standards, the creation of a European certificate for child-appropriate platforms is a promising option. Such a label—analogous to data-protection or sustainability certifications—could operate as an incentive system by linking legal compliance with visibility in the public sphere. It would foster transparency and exert market-based regulatory effects, as parents, schools and public institutions would be more likely to choose platforms with demonstrably high levels of child safety.

Sustainable regulation also requires institutional coherence. The current distribution of responsibilities—for example in Germany between federal and state levels and self-regulatory bodies—leads to uncertainty about competencies and patchy oversight. A European centre of excellence for digital child protection could pool research, monitoring and coordination functions. This body should operate similarly to the UK’s Information Commissioner’s Office and act as an interface between politics, industry and academia.

Regulation alone is not enough. Empirical evidence from Canada, the UK and Scandinavia shows that legal interventions have long-term impact only when flanked by pedagogical and societal measures [2, 28]. Digital education must therefore be established as an integral component of school curricula. It should go far beyond technical skills to address algorithmic transparency, data ethics, online communication and digital rights. Schools play a dual role here: they are venues for competence development and spaces where children are empowered to recognise and manage risks autonomously.

Parental media education also needs to be strengthened. This corresponds to EKKJ’s recommendation to strengthen the role of parents, teachers and caregivers, who play a crucial role in supporting children’s digital learning processes [11]. National programmes—modelled, for example, on Australia’s “Stay Smart Online”—could provide parents with practice-oriented training materials, online modules and counselling services to support their ability to accompany children’s digital socialisation. It is crucial to conceptualise parents not as mere control authorities but as partners in digital education.

Particular attention must be paid to digital inclusion for socially disadvantaged groups. Children from precarious socio-economic backgrounds or with special needs often rely on social media as low-threshold communication spaces [3]. Protection measures must not structurally exclude these groups. Targeted funding programmes are therefore needed to ensure equal access to safe online spaces—for instance through school-based media labs, community learning centres or dedicated investment in digital infrastructure in disadvantaged regions.

In addition, multiprofessional support structures should be expanded. Networking schools, psychosocial services and online counselling centres can increase responsiveness in cases of digital crises. A Europe-wide hotline network, similar to Canada’s Cybertip.ca, would enable rapid reporting and processing of online violence or sexual harassment.

In the long term, a fundamental shift towards contemporary digital-media education is required. Protection must not be understood primarily as control but as empowerment. Children and adolescents need learning environments in which they can develop digital agency, critical reflection and social responsibility. Studies show that young people who participate in media-educational settings share risky content significantly less often and display higher levels of digital self-efficacy [2, 15]. A European strategy that interlinks educational initiatives, technological safety and legal regulation would not only ensure protection but also lay the foundation for equitable and democratic digital participation.

In summary, the future of digital child protection lies in an integrated, evidence-based approach. Only by combining binding age verification, age-appropriate platform design and comprehensive media education can the protection of children in digital environments be realised without undermining their rights to participation, information and self-determination.

## Discussion: between protection, participation, and freedom

International developments in digital child-protection strategies reveal a fundamental tension between protection-oriented and participation-oriented approaches. Political and societal discourses often position children within a dichotomy of vulnerability and innocence, depicting them as beings in need of protection rather than as active agents [5]. This paternalistic protection discourse is mirrored in restrictive regulatory models that control access to digital spaces via age limits and prohibitions. From a child-rights and developmental-psychological perspective, however, such logic is overly simplistic: protection that precludes participation reproduces dependency and prevents the development of the very digital competences that underpin long-term safety [2, 26].

A contemporary approach must therefore account for the dialectical relationship between protection and autonomy. Empirical evidence shows that children are not merely potential victims of digital risks but at the same time active co-creators of their media lifeworlds [15]. Many adolescents have sophisticated strategies for avoiding risks, negotiating social norms and handling digital conflicts. Restrictive policy forms that ignore these competences risk disempowering children and depriving them of the very learning spaces they need to develop self-protection mechanisms. Against this backdrop, the concept of digital-media education is gaining in importance. Its aim is not only to protect children and adolescents, but also to empower them to use digital spaces safely, critically and autonomously. In contrast to reactive or deficit-oriented approaches, digital-media education understands media use as part of children’s lifeworld and as a site of social, cultural and political learning. It combines media-pedagogical and ethical perspectives with legal and technological protection mechanisms. In this respect, it aligns with international recommendations from UNICEF [32] and the Council of Europe (2022–2027), which emphasise that children’s rights in digital environments must be realised not only through protection but through empowerment.

Such a perspective requires a normative paradigm shift: away from the assumption that safety is achieved through restriction, towards an understanding of protection as enabling. This implies that children need access to digital spaces that are both safe and participatory. Age verification and design obligations can create necessary structural conditions, but their effectiveness depends crucially on being embedded within a broader pedagogical concept. Empirical studies from the UK and Canada show that long-term protective effects are considerably higher where legal regulation and educational programmes are closely interlinked [2, 28].

A second aspect concerns the socio-ethical dimension of digital participation. Restrictive protection logics tend to exacerbate social inequalities by denying access particularly to those children who rely on digital spaces to compensate for social disadvantage [3]. Child-appropriate digital policy must therefore understand inclusion and protection as complementary principles. The goal is not to reduce digital presence but to ensure its fair and safe organisation.

Moreover, the debate raises questions about the responsibility of technology providers. While traditional regulatory approaches have largely targeted parents and educational institutions, it is now widely recognised that platform design itself constitutes a central risk factor [22]. Algorithms designed to maximise engagement and emotional attachment can foster dependency and manipulative dynamics. This generates an obligation for industry to treat safety and transparency as integral aspects of product architecture—a principle that must be institutionalised within the framework of safety-by-design.

Finally, the discussion on digital child protection can be read as part of a broader societal negotiation of autonomy, control and trust. Children are growing up in a world characterised by pervasive digital connectivity, in which withdrawal from online spaces is neither realistic nor desirable. Consequently, protection must be organised not in opposition to digitalisation but within digital lifeworlds. The challenge is to develop a culture that takes children’s digital self-determination seriously while offering them reliable safety nets.

In conclusion, the effectiveness of digital protection strategies depends less on the severity of regulatory measures, but on their integration into pedagogical, social and technological contexts. Modern digital policy must therefore find the courage to move away from prohibition and instead prioritise empowerment, responsibility and participation. Digital-media education thus becomes the central guiding concept of future-oriented child-protection policy—not as a substitute, but as a necessary extension of legal regulation. It reconciles protection and freedom, prevention and participation, and marks the transition from a paternalistic to a child-rights-based understanding of digital lifeworlds.

## Data Availability

Not applicable.
